# Culture-free bacterial detection and identification from blood with rapid, phenotypic, antibiotic susceptibility testing

**DOI:** 10.1038/s41598-018-21520-9

**Published:** 2018-02-21

**Authors:** Xuyang Shi, Usha Kadiyala, J. Scott VanEpps, Siu-Tung Yau

**Affiliations:** 1 Department of Electrical Engineering and Computer Science, Cleveland State University, Cleveland, Ohio, USA; 2 Department of Emergency Medicine, University of Michigan, Ann Arbor, Michigan USA; 3 Michigan Center for Integrative Research in Critical Care, University of Michigan, Ann Arbor, Michigan USA; 4 Biointerfaces Institute, University of Michigan, Ann Arbor, Michigan USA; 5 The Applied Bioengineering Program, Cleveland State University, Cleveland, Ohio, USA

## Abstract

The current culture-based approach for the diagnosis of bloodstreams infection is incommensurate with timely treatment and curbing the prevalence of multi-drug resistant organisms (MDROs) due to its long time-to-result. Bloodstream infections typically involve extremely low (*e.g*., <10 colony-forming unit (CFU)/mL) bacterial concentrations that require a labor-intensive process and as much as 72 hours to yield a diagnosis. Here, we demonstrate a culture-free approach to achieve rapid diagnosis of bloodstream infections. An immuno-detection platform with intrinsic signal current amplification was developed for the ultrasensitive, rapid detection, identification (ID) and antibiotic susceptibility testing (AST) of infections. With its capability of monitoring short-term (1–2 hours) bacterial growth in blood, the platform is able to provide 84-minute simultaneous detection and ID in blood samples below the 10 CFU/mL level and 204-minute AST. The susceptible-intermediate-resistant AST capacity was demonstrated.

## Introduction

The current standard for diagnosis of bloodstream infections, including device associated infections (*e.g*., central line-associated bloodstream infections, prosthetic valve endocarditis), requires sequential bacterial detection, identification (ID), and antibiotic susceptibility testing (AST) via traditional blood cultures. This culture-based three-step diagnostic approach is not optimal with profound clinical implications. First, the time to result for blood cultures typically ranges from 24 to more than 48 hours^1,2^. Typical AST requires an additional 16–24 hours of culture of the isolated pathogen^3^. Understanding that early antimicrobial therapy reduces mortality in bloodstream infections, patients are given empiric, broad-spectrum antibiotics pending culture results. This one-size-fits-all use of antibiotics results in opportunistic infections, drug-related toxicities, and antibiotic resistance. The prevalence of multi-drug resistant organisms (MDROs) is poised to be one of the greatest threats to global public health as new MDROs emerge over time^4^. Each year, in the United States, over 2 million people acquire serious infections with bacteria that are resistant to one or more of the antibiotics designed to treat those infections^5^. At least 23,000 people die each year as a direct result of these antibiotic-resistant infections^5^. Many more die from other conditions that are complicated by an antibiotic-resistant infection. Second, blood cultures have low sensitivity. When bacteria in the blood are in low numbers (<10 CFU/mL) growth is sufficiently slow to produce a negative result^6–8^. Worse still, certain bacteria do not grow at all under standard culture conditions^9^. Up to 30% of prosthetic valve endocarditis is initially culture negative^10^. Third, cultures are frequently contaminated by normal skin flora^11^, which can grow rapidly and out-compete certain pathogens in the culture media. Repeating cultures to confirm contamination versus infection further extend the time to diagnosis.

A rapid diagnostic for bacterial detection, ID, and more importantly, AST would reduce exposure time to broad-spectrum antibiotics and allow for rapid de-escalation to pathogen targeted therapy. Non-culture, molecular diagnostic methods are rapidly being incorporated into standard medical microbiology laboratories. Most techniques are based on nucleic acid detection and/or amplification (*e.g*., polymerase chain reaction). However, direct detection of bacterial nucleic acid in whole blood remains a challenge. This is related to the exceedingly low concentration of bacteria in the blood in a high background concentration of human cells^12^. To date there are no FDA approved diagnostics for direct detection of bacteria in whole blood without culture pre-enrichment. Although the current molecular techniques, that still require culture enrichment, improve the time to AST with demonstrated clinical benefits including decreased length of stay and healthcare cost^13^, they all suffer the failures of blood cultures previously mentioned.

**Figure 1:**
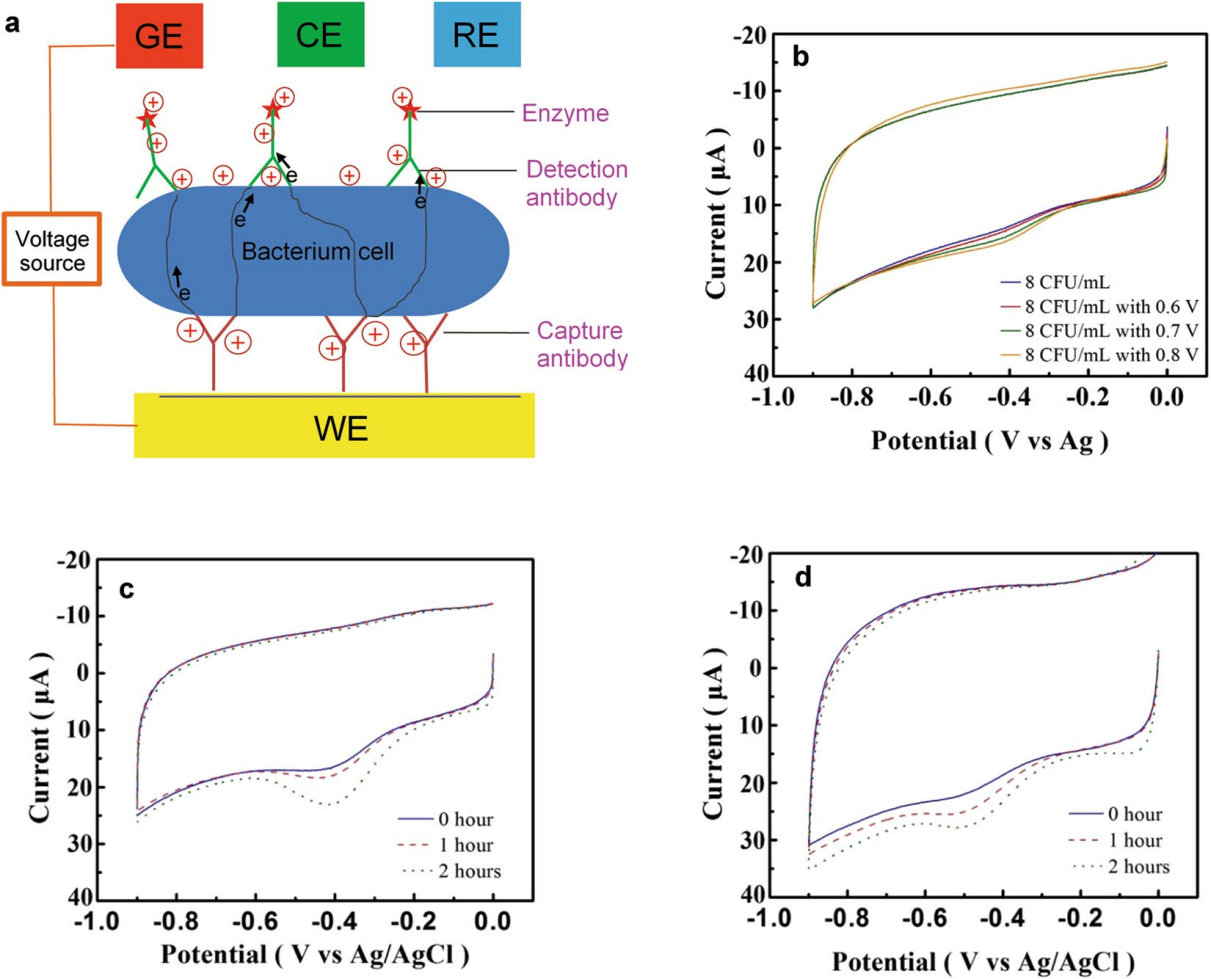
The FEED-based platform and its capabilities. (**a**) A schematic description of the FEED-based detection platform, where WE, RE, CE and GE are, respectively, the working electrode, reference electrode, counter electrode and gating electrode. The gating voltage induces negative charges on WE and positive charges on the immune complex. A gating voltage V_G_ is applied between GE and WE. (**b**) The amplification of the detection signal due to V_G_. The concentration of *E. coli* was 8 CFU/mL. Without V_G_, a weak HRP reduction peak with a height of 2.2 μA appears at −0.42 V. This peak is progressively amplified with V_G_ = 0.6, 0.7, 0.8 V. Culture enrichment was not performed. (**c**) and (**d**) Monitoring short-term bacterial growth. The HRP reduction peak, the detection signal, increases over 2 hours, indicating the growth of *E. coli*. CVs were obtained with V_G_ = 0.6 V. (**c**) shows the growth in a nutrient broth, while (**d**) without broth in blood. The *E. coli* concentrations in (c) are 9, 48, 270 CFU/mL, and in (d) are 8, 45, 128 CFU/mL.

Here, we describe a culture-free and therefore rapid method to achieve bacterial detection/ID (84 min sample-to-result) followed by rapid AST (204 min sample-to-result). The detection platform is based on the technique of field effect enzymatic detection (FEED)^14^. A detailed description of the principle of the system is available in previous publications^14,15^ and in the Supporting Information (Fig. S1 and S2). The method employs an immuno-assay platform^16,17^ for the direct detection of bacteria in samples without culture enrichment. Fig.1a is a schematic description of the detection platform. Horseradish peroxidase (HRP), a redox enzyme, is immobilized on the working electrode (WE) via a sandwich immune complex. The gating voltage, V_G_, induces an electric field at the solution-enzyme-electrode interface to reduce the tunnel barrier for electrons. Therefore, the tunnel current between the electrode and HRP, the signal current, is amplified by V_G_. The ultrasensitive, quantitative detection of bacteria provided by the platform due to signal amplification allows the direct detection of bacteria in extremely low concentration blood samples without sample processing. Specifically, we show the detection of *E. coli* directly from blood samples below 10 CFU/mL in 84 minutes. Since the detection is based on the specific immuno-reaction between bacteria and antibodies, the result of the detection also identifies the detected bacteria in a multiple-bacteria sample, and, therefore, detection and ID are simultaneously achieved. Further, the ultrasensitive detection capability of the platform facilitates the monitoring of bacterial growth in response to antibiotics over short time frames. This capability was leveraged to demonstrate rapid (204 min sample-to-result) AST by exposing susceptible and resistant strains of *E. coli* to ampicillin. The AST results provide quantitative information on the response of bacteria to antibiotics, differentiating bacteriostatic from bactericidal responses.

## Results

### Direct detection and signal amplification

The direct detection of wild-type (WT) *E. coli* was based on the amplification of the current of the HRP reduction peak, which occurs between −0.42 V and −0.52 V on the cyclic voltammogram (CV) of the detection electrode as shown in Fig. S3a. In general, when the bacterial concentration is less than 10 CFU/mL, without applying V_G_, the CV is similar to that of negative controls (NC), which are blood samples without bacteria. The CVs of NC show a weak peak at about −0.45 V as shown in Fig. S3b. The NC peak height statistically ranges between 2.0 μA and 3.0 μA, defining a band of currents for this characteristic offset. Although, this weak peak is within the potential range of the HRP reduction peak, it cannot be amplified by V_G_ as shown in Fig. S3b. Fig. 1b shows the effect of V_G_ on the detection of 8 CFU/mL of *E. coli* in whole blood. The blue CV was obtained without applying V_G_. The CV shows a weak peak with a height of 2.2 μA at −0.42 V. This peak is ambiguous since its peak height is within the NC band. However, this peak responds to V_G_. The reduction peak of the red CV, obtained with V_G_ = 0.6 V, has a current of 3.8 μA, which is unambiguously above the NC band. The red CV shows that application of V_G_ amplifies the HRP peak to facilitate the assessment of bacterial concentration. Fig. 1b shows that the amplification can be enhanced by increasing V_G_.

In general, relatively large sample volumes are needed to ensure the presence of bacterial cells for the detection of ultra-low concentration samples. In the present work, 200 μL samples were used for concentrations greater than 7 CFU/mL, implying that nominally 1–2 CFUs were immobilized on the electrode. At the lowest concentrations used in the present work, V_G_ is required to amplify the current peak in order to lift the ambiguity so that the signal is reliably detected. In general the overall sensitivity of the assay can be increased by increasing the sample volume exposed to the electrode.

### Short-term monitoring of bacteria growth

The FEED platform’s capacity to detect extremely low bacterial concentration without culture was used to monitor short-term changes in bacterial concentration due to bacterial growth. Fig. 1c shows the increasing detection signals due to the growth of WT *E. coli* in a blood sample during a 2-hour period. The initial *E. coli* concentration was 9 CFU/mL and a nutrient broth (*i.e*. Luria Bertani broth) was used to facilitate the growth. The CVs were obtained with V_G_ = 0.6 V and show a progressive increase in the HRP reduction peak current indicating bacterial growth. The ultrasensitive detection capability of the platform even allows bacterial growth to be monitored without adding nutrient broth to blood samples as shown in Fig. 1d. The signal currents are less than those with the broth due to slower growth.

### Calibration curve in blood

**Figure 2:**
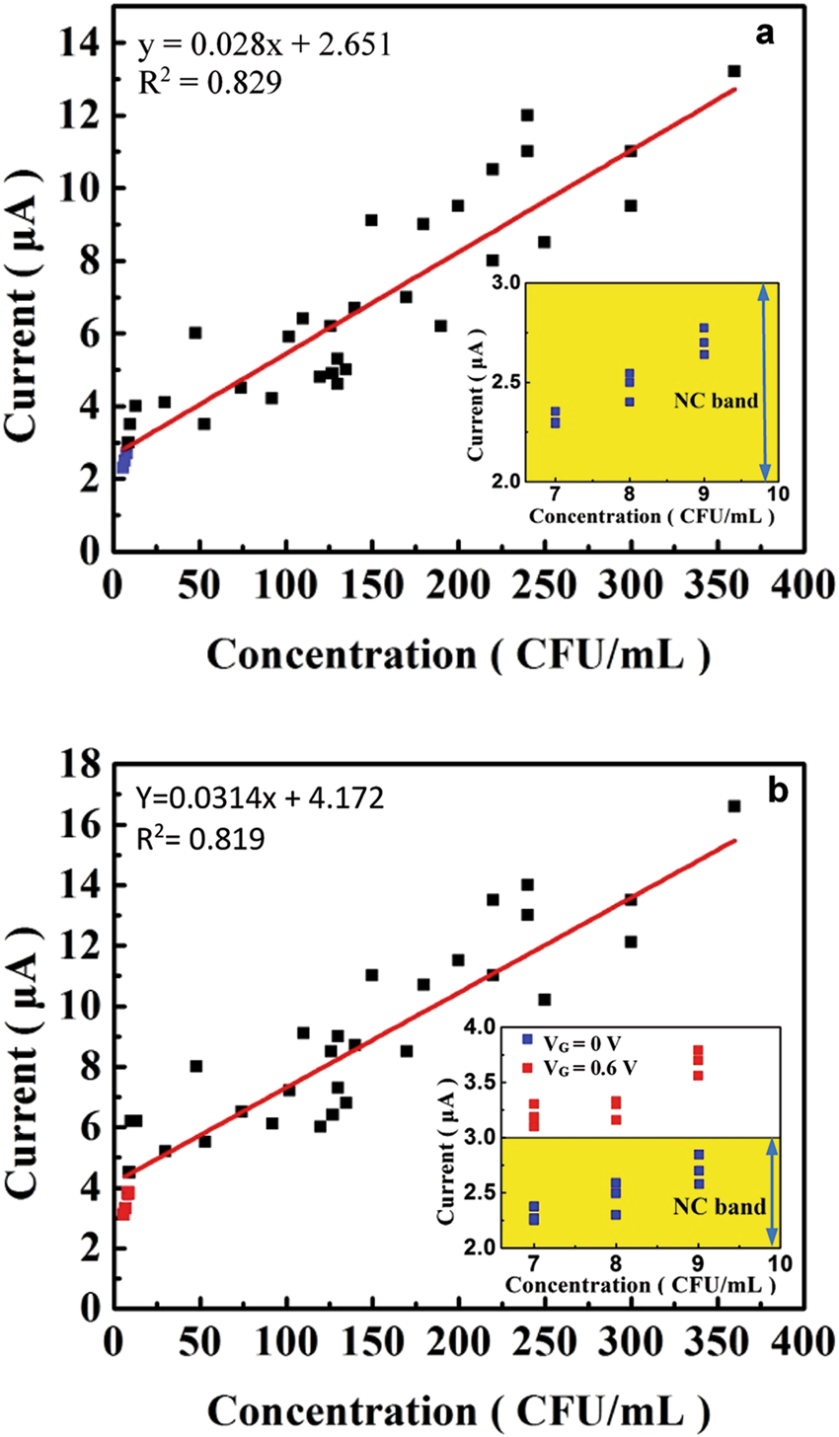
The calibration curves for *E. coli* in blood. The curve in (**a**) was obtained without applying V_G_, while the curve in (**b**) with V_G_ = 0.6 V. The two insets show the effect of V_G,_ which lifts the blue data points above the NC band.

The platform’s capacity to quantify bacteria in blood was demonstrated by generating calibration curves as shown in Fig. 2. Samples were generated by spiking whole blood with a specific inoculum of WT *E. coli*. For each nominal concentration made by diluting the inoculum, three samples were tested using the platform and culture was performed on the samples. Therefore, the curves consist of data points that correlate the measured signal currents to the actual *E. coli* concentration determined by culture and colony enumeration of spiked samples. At concentrations above 10 CFU/mL, culture yields larger discrepancies in a nominal diluted concentration. Therefore, the three identical diluted concentrations of a nominal value yields different values by culture. In the construction of the calibration curves, more than 60 measurements were performed on spiked samples (positive control) and only two measurements failed to detect a signal due to fault in the preparation of electrodes. Thus, the rate of success was >95%. Fig. 2a shows the calibration curve obtained without applying V_G_ while the curve in Fig. 2b was obtained with V_G_ = 0.6 V. The plots show an increasing trend of the signal current with increasing bacterial concentration. The trend lines were obtained using linear regression. The application of V_G_ = 0.6 V shifts the trend line in Fig. 2b upwards compared with that in Fig. 2a.

The three blue data points in Fig. 2a show the signals produced by concentrations below 10 CFU/mL. These data points are within the band of NC values as shown in the inset. These points were not used to determine the regression line. The inset of Fig. 2b shows that the application of V_G_ elevates the blue points above the NC band so that the points below 10 CFU/mL become part of the calibration curve as indicated by the three red circles. The curves can be used for assessing bacterial growth during AST.

### Detection and identification

**Figure 3:**
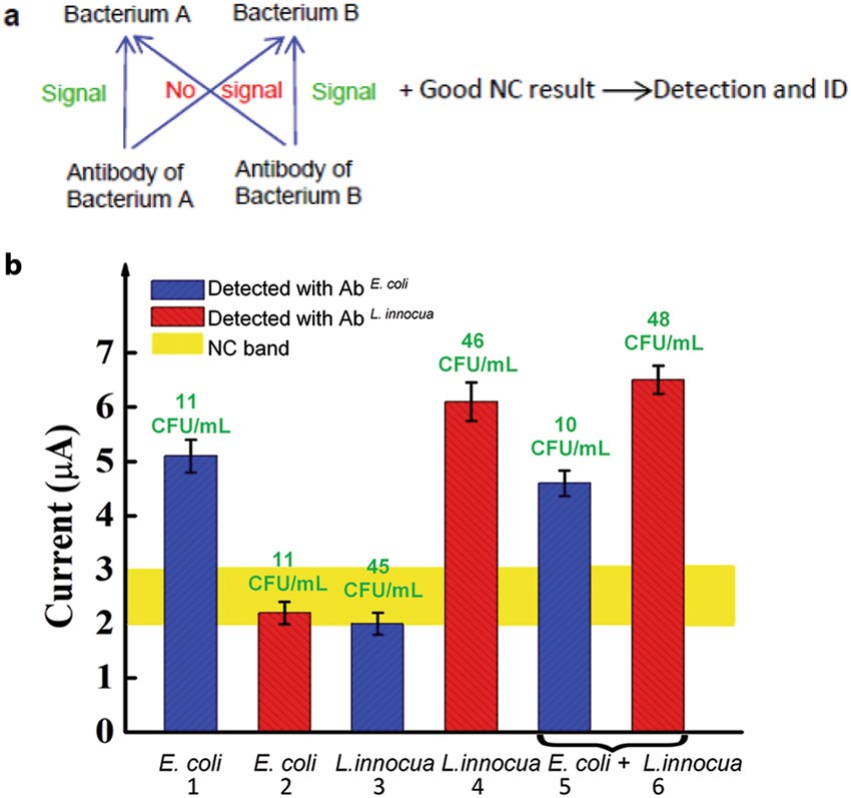
Simultaneous detection and ID using the FEED-based platform. (**a**) A paradigm for the simultaneous bacterial detection and ID of two bacterial species in the same sample. (**b**) Detection signals of samples containing *E. coli, or L. innocua* or both. The bacterial concentration obtained by culture (the green prints) is shown above each bar. The numbered bars indicate the detection of specific bacterium detected with specific antibodies. Error bars are based on three replicates.

The platform’s capacity for direct detection of *E. coli* in low concentration samples without culture pre-enrichment indicates the feasibility of using the platform to simultaneously achieve detection and bacterial ID. Fig. 3a shows the conditions that validate such a paradigm in the two-bacterial species case. The detection of Bacterium A using its antibody should generate a signal. The platform’s null signal generated by Bacterium B using the antibody of Bacterium A indicates selectivity for Bacterium A. If similar results are obtained for the converse (*i.e*., detection signal for Bacterium B and null signal for Bacterium A using antibody against Bacterium B), with acceptable negative control results, the platform is able to detect and identify the two species in the same sample in a parallel process. This paradigm allows the platform to simultaneously detect and identify multiple bacteria in the same sample.

WT *E. coli* and *L. innocua* were used to demonstrate this paradigm. Blood samples spiked with each or both bacterial species were used. The actual bacterial concentration was confirmed by plating and colony enumeration. The platform detection and standard plate culture results are shown in Fig. 3b. The bacterial concentration obtained by plate culture is shown above each bar. In the *E. coli-*only samples (Bar 1 and Bar 2), signal is detected above the NC band using the *E. coli* antibody but not the *L. innocua* antibody. This indicates the platform’s selectivity for *E. coli* against *L. innocua*. Likewise, only the *L. innocua* antibody demonstrates signal above the NC band for the *L. innocua-*only samples (Bar 3 and Bar 4), demonstrating selectivity for *L. innocua* against *E. coli*. In samples containing both *E. coli* and *L. innocua*, both antibodies show a signal. Using an *E. coli* detection electrode, a signal of 4.7 μA was measured from a sample containing nominally 10 CFU/mL *E. coli* and 40 CFU/mL *L. innocua* whereas culture indicates that there are 10 CFU/mL of *E. coli* in the sample (Bar 5). Using an *L. innocua* detection electrode yields a signal of 6.4 μA whereas culture shows 48 CFU/mL in the sample (Bar 6). Note that these signals are nearly identical to the signals from samples that contain only one bacterial species. This indicates almost no detection interference between the two species in these samples. Therefore, the results in Fig. 3b, with NC results like that shown in Fig. S3b, indicate that *E. coli* and *L. innocua* were both identified and quantitatively detected. The time used to perform the detection and identification in the two-species sample was 84 min as described in Methods. As a note, during routine use, the results of the first four columns will be established as a property of the platform and those corresponding measurements will not be made for each individual sample.

### AST

AST is a critical step for clinical treatment of infectious diseases^18^. AST evaluates the ability of a bacterium to replicate in the presence of a specific antibiotic^19^. AST allows clinicians to treat infections with the narrowest-spectrum antibiotic that is most effective against the causative pathogen. AST is typically performed by disk diffusion, gradient diffusion, or agar/broth dilution^20^. Standardized inoculums of culture isolates are needed to perform these tests, and the tests require 16–24 hours from isolated pathogen to results, making them time- and labor-consuming studies^21^. The results of these tests, except for that of the gradient diffusion test and the broth dilution test, are semi-qualitative, in that the tests provide only a category of susceptibility (*i.e*., susceptible, intermediate or resistant)^19^.

**Figure 4:**
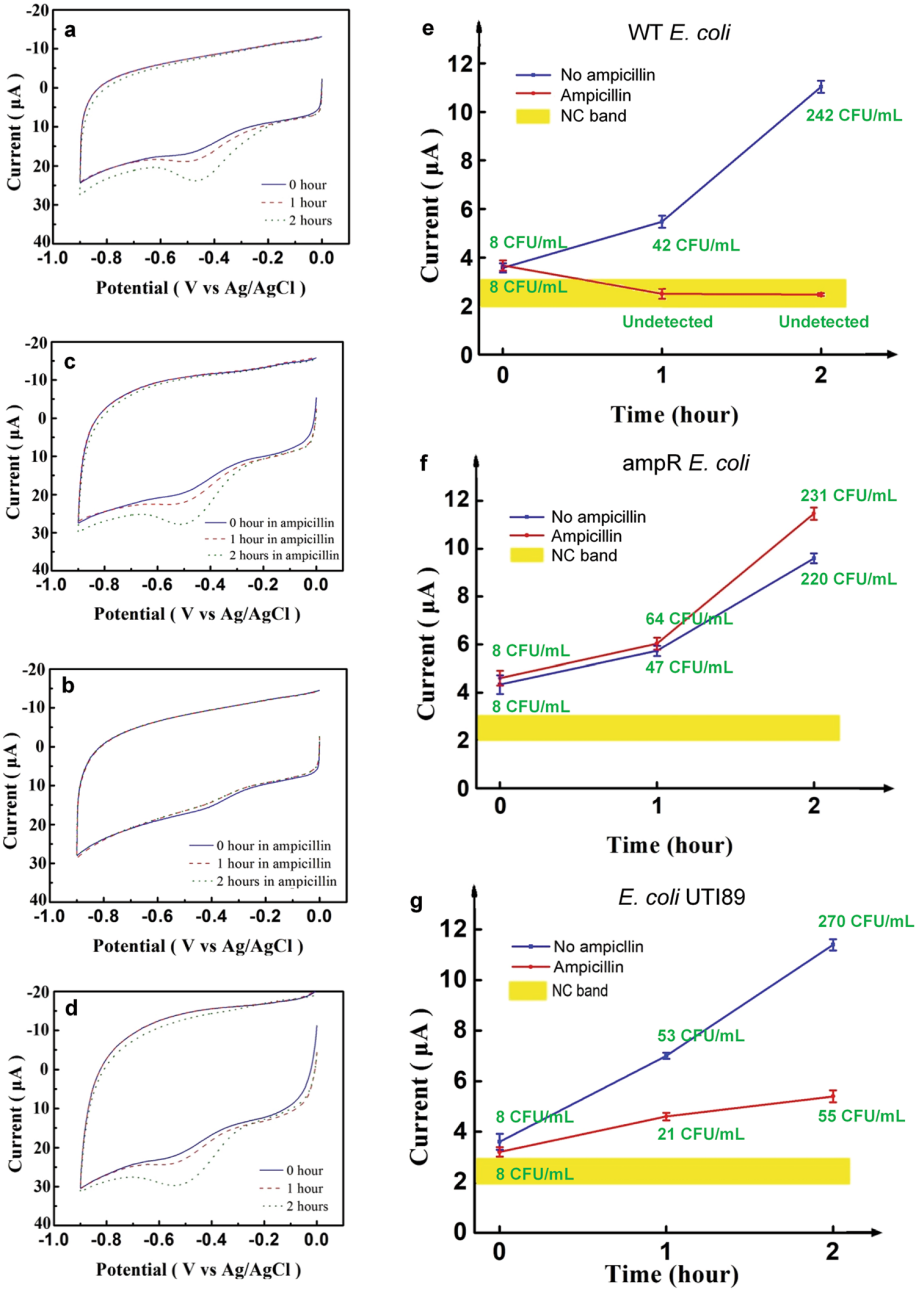
AST results. (**a**–**d**) The effect of ampicillin on the growth of the two strains of *E. coli* monitored using the platform is shown in (**a**) WT *E. coli* without ampicillin, (**b**) WT *E. coli* in 8 μg/mL of ampicillin, (**c**) ampR *E. coli* in 8 μg/mL of ampicillin, and (**d**) ampR *E. coli* without ampicillin. A nutrient broth was used in (**a**–**d**). (**e**) and (**f**), respectively, summarize the 2-hour growth profiles of WT *E. coli* and ampR *E. coli* with and without ampicillin. **(g)** The growth profile of UTI89, an intermediate resistance strain of *E. coli*, in 8 μg/mL of ampicillin.In (**e**)-(**g**), the bacterial concentration determined from standard plating and colony counting (the green prints) is shown for each data point. Error bars are based on three replicates.

With its capacity to monitor short-term bacterial growth, the platform is able to provide AST on bacterial samples that have been exposed to antibiotics for 2 hours. To illustrate this capacity, the growth responses of the WT *E. coli*, which is pan-susceptible, and an ampicillin-resistant (*i.e*., ampR *E. coli*) strain to ampicillin were detected using the platform. Fig. 4a and b show the CVs of a blood sample with 8 CFU/mL of WT *E. coli* with and without ampicillin, respectively, growing in a nutrient broth over 2 hours. The progressive increase in the peak current in Fig. 4a indicates the continued growth of the WT *E. coli*. The minimum inhibitory concentration (MIC) of ampicillin for this strain was measured to be 3 μg/mL of ampicillin (see Methods). Fig. 4b shows the CV of the WT *E. coli* after being exposed to 8 μg/mL of ampicillin, about 2.7 times the MIC of this strain. The exposure to ampicillin makes the 1-hour and 2-hour CVs similar to that of the NC, indicating undetectable bacterial concentration in the sample. In other words, the concentration of bacteria actually decreased in the presence of ampicillin. This is expected since ampicillin is a bactericidal antibiotic that kills bacteria, leading to their lysis. Thus, Fig. 4b shows the bactericidal effect of the antibiotic at this concentration. The CVs in Fig. 4c show the continued growth of ampR *E. coli* after being exposed to 8 μg/mL of ampicillin. The observed growth is similar to that shown in Fig. 4a. Fig. 4d shows the CVs of ampR *E. coli* in the absence of ampicillin and the CVs are similar to those in Fig. 4c. These results confirm the resistance of the ampR *E. coli* strain to ampicillin. Fig. 4e and f summarize the results of these AST measurements. In this format, AST can be completed in 204 minutes (120 min for AST + 84 min for detection). The detected bacterial growth was confirmed using standard plating and colony enumeration. Note that the detection signal for WT *E. coli* grown in ampicillin is within the NC band. Fig. 4e and f also indicate that even at 1-hour, the WT and resistant strains can be differentiated, leading to 144 min (60 min for AST + 84 min for detection) AST. To confirm that the platform is able to detect the effect of antibiotics on high bacterial concentration samples, the AST procedure was performed on *c.a*. 500 CFU/mL *E. coli* samples and similar trends were observed (See Fig. S4). Fig. 4g shows the AST profile of another *E. coli* strain, UTI89, whose MIC for ampicillin is 10 μg/mL. The profile shows similar growth to the WT strain (Fig. 4e and d**)** in the absence of ampicillin. Exposure to 8 μg/mL of ampicillin has reduced but not completely inhibited the growth of this strain. Therefore, Fig. 4g demonstrates the platform’s capacity for intermediate MIC.

**Figure 5:**
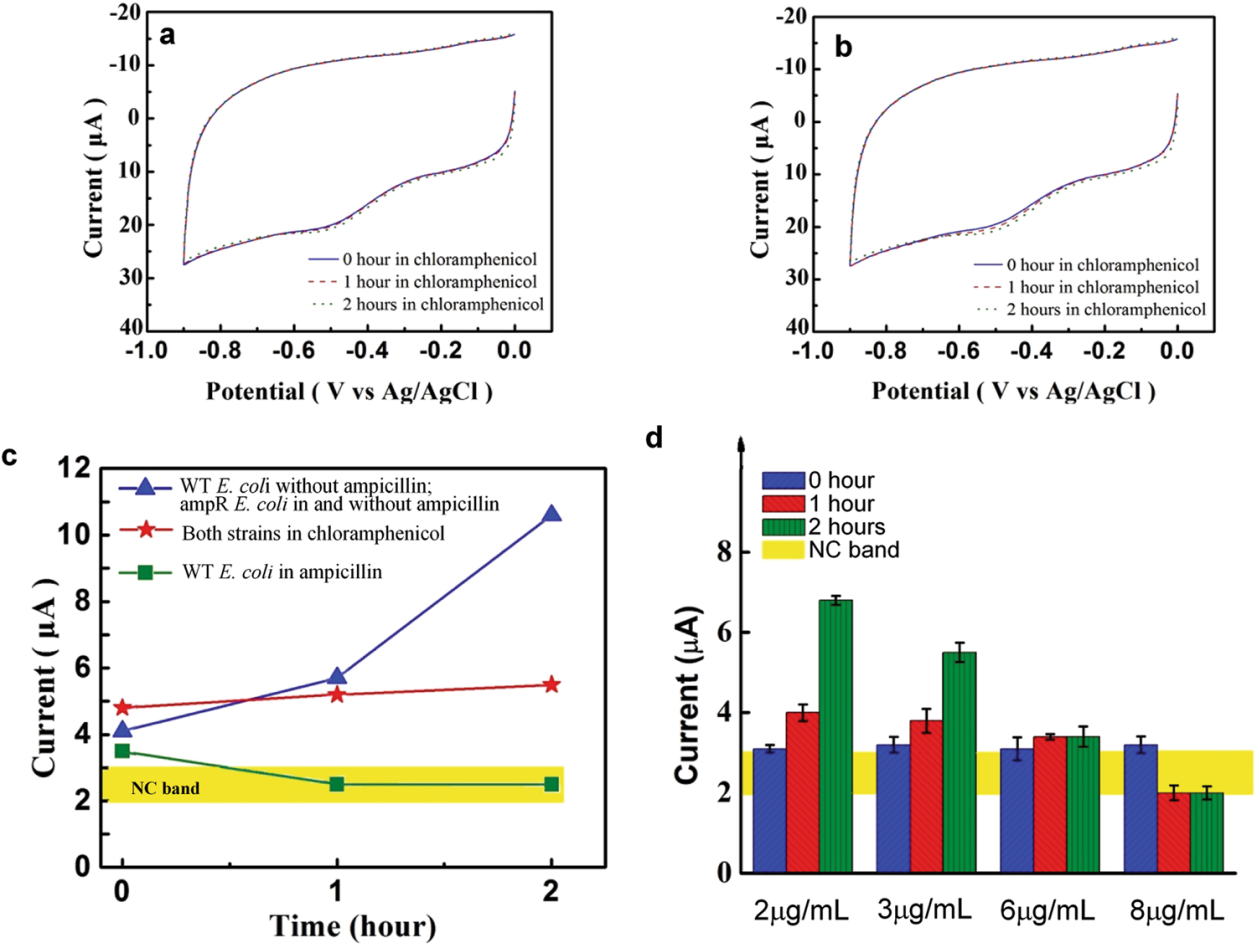
Quantitative AST of *E. coli* strains to ampicillin and chloramphenicol determined over 2 hours. The CVs in (**a**) and (**b**), respectively, show the responses of WT *E. coli* and ampR *E. coli* to their respective MICs of chloramphenicol (4 μg/mL and 6 μg/mL). A nutrient broth was used in the growth. (**c**) The signal-time profiles of the *E. coli* strains for ampicillin and chloramphenicol. The responses of the two strains to the antibiotics are characterized by three distinct groups according to the nature of the antibiotics. The yellow band indicates the NC band. (**d**) The platform reveals detailed information on the growth of 8 CFU/mL WT *E. coli* about 3 μg/mL ampicillin, the MIC determined by standard culture. Error bars are based on three replicates.

In contrast to ampicillin, chloramphenicol is a bacteriostatic antibiotic that inhibits bacterial growth without killing bacteria. AST of chloramphenicol on the two strains of *E. coli* was also performed. Fig. 5a and b display the CVs obtained in monitoring the growth of the two strains of *E. coli* exposed to their respective MICs (4 μg/mL and 6 μg/mL) of chloramphenicol. The CVs show that the platform detects the initial bacterial concentration of 9 CFU/mL. However, the platform detects almost no growth in response to chloramphenicol, indicating the bacteriostatic nature of the antibiotic. A comparison of AST with chloramphenicol versus ampicillin is shown in Fig. 5c. This demonstrates that the platform not only detects antibiotic susceptibility but further provides information on bactericidal vs. bacteriostatic mechanism of the antibiotic, all of which can be made available in *c.a*. 200 minutes or less. Specifically, the blue line indicates normal growth or resistance; the red line indicates growth inhibition or a bacteriostatic antibiotic; and the green line indicates lysis or bactericidal antibiotic. Fig. 5d shows that the platform provides additional resolution over standard MIC determination. This is a feature of its capacity to monitor bacterial growth under varied antibiotic conditions. The concentration of ampicillin was varied about the MIC value for the WT *E. coli* determined using standard broth microdilution (see Methods). Specifically, the FEED platform demonstrated continued growth of *E. coli* at the MIC albeit reduced compared to lower concentrations. It is not until we reach *c.a*. 2.7x the MIC that bactericidal effects are seen. Regardless, this information is available in *c.a*. 2 hours.

### Demonstration of rapid, direct AST

The present practice of AST requires bacteria isolates from clinical samples. To perform bacteria isolation, the bacterial colonies are first spatially separated by plating. Then, a variety of microbiological and biochemical techniques are used to assign the identities of the separated bacterial colonies. An inoculum is subsequently standardized to a specific concentration for a susceptibility test. Previously, conventional AST methods have been applied to unprocessed samples. The main criticism of such direct susceptibility test (DST) is the non-standardized sample and mixed cultures, which may lead to inaccurate results^22,23^. Nevertheless, numerous studies conducted over the past two decades indicate the applicability of DST in the diagnosis of infections^24–27^.

**Figure 6:**
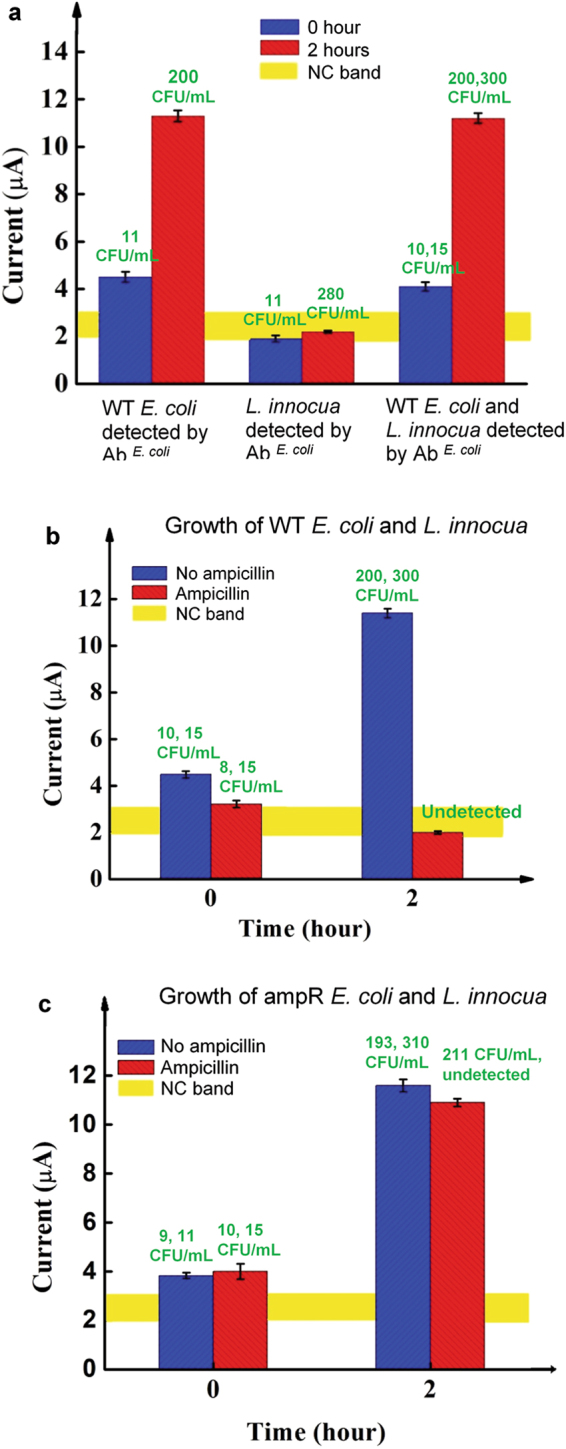
Demonstration of RAST for a period of two hours. (**a**) Selective detection of the growth of *E. coli* in an *E. coli*-*L. innocua* blood sample is indicated by the platform’s detection signal and the bacterial concentration from culture in unit of CFU/mL(the green prints). The first and second groups of data were obtained with blood samples spiked with *E. coli* or *L. innocua*, respectively. The last group was obtained from samples spiked with *E. coli* and *L. innocua*. The culture results are shown in the same order. (**b**) and (**c**), respectively, show the platform’s detection signals of WT *E. coli* and ampR. *E. coli* in the presence of *L. innocua*. The detection was performed with *E. coli* specific electrodes. The culture results of *E. coli* and *L. innocua* are shown above each bar in the same order. (**b**) and (**c**) show the AST of *E. coli* for ampicillin in the presence of *L. innocua*. Error bars are based on three replicates.

The 204-minute sample-to-result ampicillin susceptibility measurements indicate the feasibility of rapid AST (RAST), which measures the response of a bacterium in an unprocessed sample (without bacterial isolation and sample standardization) to typical antibiotics. This application requires that the platform exhibits selectivity for the target bacterium. The selectivity of the platform in the detection of *E. coli* was demonstrated and the results are shown in Fig. 3. The platform’s capacity to perform selective monitoring of bacterial growth was demonstrated by mixing WT *E. coli* with *L. innocua* in whole blood and using the detection electrodes prepared to detect *E. coli* to monitor the 2-hour growth of the mixed sample. The results are shown in Fig. 6a. The 2-hour growth of *E. coli* with initial concentration of 11 CFU/mL was monitored using the platform and shows a predictable increase. Again, the growth was confirmed by standard plating and colony enumeration.

The detection signals of *L. innocua* are within the NC range while culture shows observable bacterial growth. The detection signals for the sample containing both species are similar to that obtained by *E. coli* alone, despite the fact that culture shows growth in both species. These results clearly demonstrate that, at extremely low concentrations, the presence of the fast growing *L. innocua* does not interfere with the specific detection of *E. coli* and therefore indicate the platform’s selective monitoring of the growth of *E. coli*.

Based on this selectivity, the platform was used to demonstrate RAST. The same procedure as that used in Fig. 4 was applied to whole blood samples containing both *E. coli* and *L. innocua*. The AST results obtained using *E. coli* specific electrodes are shown in Fig. 6b,c. The results clearly demonstrate the capacity of the platform to monitor the short-term bacterial growth of a selected species in the presence of another species from concentrations *c.a*.10 CFU/mL and provide a 204-minute sample-to-result AST.

To perform RAST, the platform directly quantifies the changes in bacteria concentration (CFU/mL) after exposure to antibiotics. The concentrations were obtained immediately after the measurements were completed by using the calibration curve of the platform. For example, the *E. coli* concentrations converted from the 1-hour and 2-hour current measurements shown in Fig. 4 using the calibration curve in Fig. 2b compare reasonably well with the culture results. The immune reaction between a bacterium and its antibody is highly specific and therefore provides isolation and selection of bacteria. Thus, RAST produces a direct and quantitative result on a particular bacterium in the sample.

## Discussion

The FEED-based immuno-assay platform is capable of detecting bacterial concentrations (without culture pre-enrichment) below 10 CFU/mL in whole unprocessed blood in 84 min and monitoring bacterial growth at this level with selectivity. As a result, we have demonstrated two applications of the platform, namely, simultaneous detection-ID of bacteria and RAST. Proof-of-concept of the simultaneous detection-ID process was illustrated by the detection of *E. coli* in a whole blood sample containing *E. coli* and *L. innocua*. The process was completed in 84 min. With the current standard of care, this process typically requires first a 24–48-hour culture, followed by spatial separation of the bacteria colonies in the culture and assignment of the identities of the separated bacteria.

We also provided a proof-of-concept example of RAST by performing a 204-min sample-to-result AST using the platform. The results indicate that the platform is able to provide quantitative assessment of the susceptibilities of two strains of *E. coli* to ampicillin and chloramphenicol after 2 hours of exposure to the antibiotics. In fact, the 1-hour measurements already indicate antibiotics susceptibility; the 2-hour measurements only confirm the 1-hour results. This observation implies the possibility of 144 min (60 min for AST + 84 min for detection) sample-to-result AST. Furthermore, the AST on the two strains of *E. coli* in the presence of *L. innocua* demonstrates the selectivity of the platform and indicate the feasibility of direct AST on samples without requiring bacterial isolation and sample standardization. The 204-minute AST time is compared with that of 16–24 hours for current culture-based AST methods. The significantly shortened diagnosis time will allow clinicians to use narrow spectrum antibiotics much sooner than previously required to treat infections with significantly improved efficacy.

Among current AST methods, only the broth dilution and gradient diffusion tests provides truly quantitative information, *i.e*. MIC, while others report only a semi-quantitative result of susceptible, intermediate, and resistant. The precision of the broth dilution method is plus or minus 1 two-fold concentration caused by the errors of the antibiotics dilution procedure^19^. The MIC result provided by the broth dilution method is determined by the turbidity of the sample. We have, using the platform, studied the MIC of ampicillin on WT *E. coli* and found a “grey zone” about the MIC value obtained by dilution as shown in Fig. 5d. Therefore, the platform provides more detailed quantitative information on susceptibility. In performing RAST, the platform directly measures the bacteria concentration in the sample to report quantitative susceptibility results. The immune reaction between a bacterium and its antibody is highly specific and therefore provides the isolation and selection of bacteria. Thus, standardization of sample bacteria isolation is not required. RAST produces a direct and quantitative result on a particular bacterium in the sample and can provide information over the entire spectrum from resistant to susceptible. However, in the case of intermediate resistance, criteria for discriminating intermediate resistance must be developed from the signal vs. time profile (see Fig. 5c).

Going forward, we envision a test kit based on the platform described here that consists of a set of detection electrodes, which are individually immobilized with the antibody of one of the of the most common pathogens. Detection and simultaneous ID of multiple bacteria can be performed based on the extension of the paradigm described in Fig. 3 (a). The envisioned ultimate RAST will be realized using an integrated array of detection electrodes. Any given sample will be incubated in different antibiotics at six concentrations. The processed samples will be tested using the platform at 0.5, 1, and 2 hours to generate a signal versus time profile. This profile will then be used to define antibiotic susceptibility. The RAST can be performed together with the detection- ID step or it can be used entirely by itself for the determination of susceptibility. Compared with conventional AST method, the platform offers several important advantages, including ultrasensitivity, fast time-to-result, quantitative description of susceptibility, and differentiation of bacteriostatic vs. bacteriocidal effects. While the FEED-based platform suggests a new approach for rapid diagnosis and treatment of infections, it can also be used as a rapid method to assess the effect of new antibiotics. The platform’s capacity to monitor short-term (*e.g*. 1-hour) bacterial growth can also be used to determine whether the bacteria in the sample are viable. Finally, the fact that the platform is able to monitor short-term bacterial growth without the addition of growth media to the sample will simplify the operational workflow of the assay when deployed in a clinical laboratory. Other implied advantages of the platform include point-of-care (POC) use, low cost provided by the use of screen printed electrodes (SPE), ease-of-use if the SPE is operated by a hand-held meter and reduced labor.

A potential weakness of this platform lies in the availability of antibodies. Thorough search of antibody manufacturers suggests that antibodies for the most commonly occurring pathogens are already commercially available. Future development of this platform will indeed require extensive quality assurance of each antibody pair for a given pathogen or strain. For those pathogens/strains without commercially available antibodies, development and testing of new antibodies will be required. Nevertheless, the possibility of detecting several strains using one antibody system is demonstrated by the AST of *E. coli* UTI89 as shown in Fig. 4g and AST on *E. coli* MG1655 (MIC 4 mg/μL) in Fig. S5.

## Methods

### Bacterial strains and plasmids

All bacterial strains were obtained from American Type Culture Collection (ATCC). *E. coli* – wild type or WT (ATCC 25922) is a Clinical Laboratory and Standard institute (CLSI) control strain for antimicrobial susceptibility testing. The ampR *E. coli* was made by transforming the ATCC 25922 strain with the pGLO plasmid containing green fluorescent protein (GFP) gene, the *araC* gene for regulation of GFP expression, and an ampicillin resistance gene (*ampR*). Transformation was performed by heat shock as described in in the Supporting Information ( Fig. S6 and S7). *L. innocua* (ATCC 33090) is a quality control strain for the detection of *Listeria*. Glycerol stocks of all strains maintained at −80 °C were plated on Luria Bertani (LB) agar, cultured overnight at 37 °C and stored at 4 °C. Two additional *E. coli* strains, UTI89 (MIC 10 μg/mL) and MG1655 (MIC 4 μg/mL) were also used for AST measurements^28^.

### Reagents and sample preparation

For *E. coli* detection, *E. coli* serotype O/K polyclonal antibody (Thermo Fisher Scientific, PA1–7213) was used as the capture antibody and horseradish peroxidase (HRP)-conjugated *E. coli* serotype O/K polyclonal antibody (Thermo Fisher Scientific, PA1-73030) was used as the detection antibody. For the detection of *L. innocua*, a commercial polyclonal antibody (KPL, 01-90-90**)** against *Listeria* was used for both the capture antibody and the detection antibody. The detection antibody was conjugated to HRP using a commercial conjugation kit (84-01-01, KPL). De-identified whole human blood from healthy donors was used to engineer samples inoculated with bacteria (Valley Biomedical, Winchester, VA).

Ampicillin (A5354) and chloramphenicol (C1919-5G) were purchased from Sigma. The MIC for each combination of antibiotic and bacterial strain was confirmed by standard broth microdilution using a 96-well plate, 10^6^ cells/ml were inoculated into increasing concentrations of ampicillin or chloramphenicol. After 16-hour incubation at 37 °C, the MIC was identified as the lowest concentration with no visible bacterial growth. The MIC of ampicillin was 3 μg/mL for WT *E. coli*. The *ampR E. coli* grew at all concentrations tested. The MIC of chloramphenicol for the WT and *ampR E. coli* were 4 μg/mL and 6 μg/mL, respectively. The nutrient medium for growth studies was LB broth.

Single colony inoculates were grown in LB broth under aerobic conditions to an optical density at 600 nm (OD_600_) of 0.4–0.8. An aliquot of this culture was diluted and spiked into a known volume of whole blood. Samples were then serial diluted in whole human blood to obtain samples of progressively decreasing bacterial concentrations. Final concentration of samples was determined by serial dilution, plating and colony counting. Whole blood without bacteria was used as a negative control (NC). For time-growth based studies an aliquot of a given sample was removed and split. Half was analyzed on the FEED platform; the other half was used for serial dilution and standard plating for colony enumeration. The remainder of the sample was either placed in LB broth or not and incubated at 37 °C. Aliquots were removed at 1 hour and 2 hours and simultaneously processed as above.

### Detection electrodes

Screen printed electrodes (SPEs) were used as the detection electrode for low-cost, disposable POC use. Commercial SPEs were purchased from Pine Research Instrumentation (Durham, NC). Its working electrode is a 4 mm × 5 mm square carbon electrode. The working electrode (WE), silver reference electrode (RE) and the carbon counter electrode (CE) are fabricated on the top side of the SPE. The dimensions of the SPE are 6.1 cm × 1.5 cm × 0.036 cm. The carbon WE was modified with a composite of carbon nanotube, Nafion and glutaraldehyde. Briefly, a mixture of single-walled carbon nanotubes (0.2% g/mL of nanotubes in 99% dimethylformamide), Nafion (0.5 wt% in ethanol and water) and glutaraldehyde (3% in water) were deposited on the carbon WE until the composite became dried. The volume ratio of the three substances was 1:1:3. An example of the modification procedure is described in an earlier publication^29^. To construct the detection electrode, the capture antibody was immobilized on the modified working electrode by an overnight incubation at 4 °C followed by washing the electrode with de-ionized (DI) water.

### Detection platform

The FEED system, described in Fig. S1, is a conventional three-electrode electrochemical cell modified with an insulated gating electrode. The cell contains phosphate buffer saline (PBS) and is driven by an electrochemical potentiostat. An external voltage V_G_ is applied between the gating electrode and the working electrode, upon which a redox enzyme is immobilized. V_G_ modifies the charge distribution at the solution-enzyme-electrode interface to induce an electric field which penetrates the enzyme to reduce its tunnel barrier (height of potential energy profile). Therefore, the tunnel current between the electrode and the enzyme can be amplified. Fig. 1(a) is a schematic description of the FEED-based platform. Horseradish peroxidase (HRP), a redox enzyme, is immobilized on the WE via the sandwich immune complex. V_G_ modifies the charge distribution at the solution-enzyme-electrode interface to induce an electric field which penetrates the immune complex to reduce its tunnel barrier. Therefore, the tunnel current between the electrode and HRP can be amplified. The platform used in the present work combines FEED with mediator-less electrochemical immuno-sensing technique, in which the immune complex is formed on the detection electrode and the detection antibody is conjugated with the enzyme^15,16^.

In the present work, a commercial electrochemical potentiostat (CHI 660 C Work Station) was employed to drive the cell and a piece of 1 mm-diameter copper wire coated with plastic was wound around the detection electrode and used as the gating electrode. CVs were generated using the potentiostat. The reduction peak current of HRP shown in the CVs of the SPE was used as the detection signal. Since no diffusional mediators and H_2_O_2_, which is the substrate of HRP, were used in the operation of the detection system, the detection method embodies a reagent-less approach. Different values of V_G_ between 0.1–0.6 V were tested. A DC power supply was used to provide V_G_.

### Detection procedure

Detection was performed directly on whole blood samples at room temperature without performing culture enrichment. The detection procedure consisted of a 50 min incubation of the WE of the SPE with 200 μL of the sample, which was confined in a Teflon cylinder on top of the WE. The incubation was followed by washing the electrode with DI water for 1 min. Then, the electrode was incubated with a solution containing the detection antibody for 30 min followed by a 1-min washing with DI. The sandwich immune complex formed on the WE and the SPE was ready for detection measurements. The detection was based on the amplification of the HRP reduction peak current, and the peak height was measured by drawing a baseline. CVs showing the typical HRP reduction peak produced by blood samples spiked with bacteria and the typical electrode response to NC samples are displayed in Figure S3 (a) and (b), respectively. The time required to generate the CVs for a detection electrode was 1.5 min. The detection on negative controls has a rate of success of 100% based on 10 plus measurements.

## Electronic supplementary material

Supplementary Information
