# Supplementary material for: Culture-free bacterial detection and identification from blood with rapid, phenotypic, antibiotic susceptibility testing
Source: Sci Rep. 2018 Feb 21;8:3416. doi: 10.1038/s41598-018-21520-9 (PMC5821834; doi:10.1038/s41598-018-21520-9)
Supplement: Supplementary file 1 — Supplementary Information [file 41598_2018_21520_MOESM1_ESM.pdf]

Supplementary Information

Culture-free bacterial detection and identification from blood with rapid, phenotypic, antibiotic susceptibility testing

Xuyang Shi[1], Usha Kadiyala[2, 3, 4], J. Scott VanEpps[2, 3, 4*], and Siu-Tung Yau[1,5*]

1. Department of Electrical Engineering and Computer Science, Cleveland State University, Cleveland, Ohio, USA
2. Department of Emergency Medicine, University of Michigan, Ann Arbor, Michigan, USA
3. Michigan Center for Integrative Research in Critical Care, University of Michigan, Ann Arbor, Michigan, USA
4. Biointerfaces Institute, University of Michigan, Ann Arbor, Michigan, USA
5. The Applied Bioengineering Program, Cleveland State University, Cleveland, Ohio, USA

* Correspondence should be addressed to S.-T.Y.(s.yau@csuohio.edu) and J.S.V. (jvane@med.umich.edu).

1. FEED system

Field effect enzymatic detection (FEED)[1] is the basic detection method used to construct the proposed platform. Redox (electroactive) enzymes are used as sensing elements for biosensors. The isolation of the active site of redox enzymes by polypeptides causes reduced electron transfer between the enzyme and the sensing electrode, creating a fundamental limit to the sensitivity and therefore the detection limit of amperometric biosensors[2]. **Figure S1a** shows the FEED system. It consists of a conventional three-electrode electrochemical cell with a cell potential V_{cell} connected between the working electrode and the reference electrode. The cell contains an electrolytic solution. An electric double layer is present near the surface of the working (sensing) electrode. The cell is modified with insulated gating electrodes for applying a gating voltage V_G between the gating electrode and the working electrode, upon which a redox enzyme is immobilized. V_G modifies the interfacial charge distribution. When V_G is positive, additional negative charges are induced on the working electrode and additional positive ions from the solution are induced at the solution-enzyme-electrode interface. **Figure S1b** depicts the net interfacial charge distribution. Some positive ions are able to set up electric fields in the presence of the transferring electrons residing at the enzyme's active sites (the red circles). Quantum tunneling of electrons from the active site through the polypeptide barrier to the electrode gives rise to the signal current[2]. The energy barrier can be lowered by an electric field so that the tunneling rate is enhanced[3,4]. **Figure S1c** shows that the induced fields lower the height of the tunnel barrier and therefore increase the signal current. The result of this process is an amplified signal current, an intrinsic property of the system. FEED has been demonstrated by the detection of glucose, using glucose oxidase as the sensing element[1]. The V_G-controlled amplification effectively

lowered the glucose detection limit from the milli molar (10^{-3} M) range obtained with the conventional enzymatic biosensing approach to the pico molar (10^{-12} M) range.

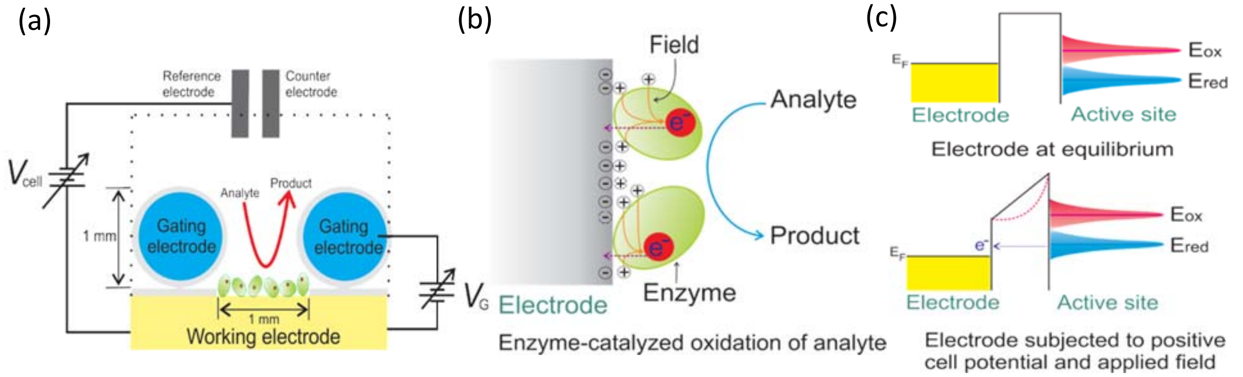

Figure S1 (a) Cross-sectional view of the FEED setup. Each elliptical structure represents an enzyme molecule. The enzyme's active center is indicated by the smaller circle within a molecule. The gating electrodes are represented by the circular structures, which consist of a copper wire (the blue circles) and a thin layer of insulator (the shaded shells). V_G is used to produce an electric field at the interface between the sample solution and the working electrode. (b) Conceptual description of enzyme-electrode interface. The red dot is the active site of the enzyme. The induced charges are indicated. The induced charge set up the field within the enzyme. The enzyme catalyzes the oxidation of the analyte, resulting in electrons e^- transferred from the active site to the electrode. (c) The effect of the V_{cell} and that of the induced electric field is depicted using the interfacial electron energy profile.

The platform incorporates FEED with an immuno-sensing methodology, whose most essential component is the immune complex. **Figure S2** shows the capture antibody-bacteria-detection antibody (Ab^c-bacteria-Ab^d) sandwich immune complex, in which the enzyme used as the sensing element is conjugated to Ab^d. The enzyme immobilized on the FEED electrode shown in **Figure S1a** is replaced by the sandwich immune complex. In addition to the intrinsic signal amplification provided by FEED, the platform also features a mediator-less operation: the transfer of electrons between the enzyme and the electrode occurs via the complex without using diffusive mediators.

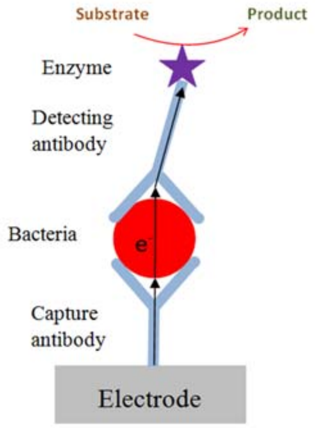

Figure S2 The sandwich immune complex formed on an electrode.

2. Characteristic cyclic voltammograms (CV) of the detecting electrode

The detection signal for the FEED platform is based on CVs of the detection electrode. Figure S3 (a) shows the CVs of an electrode with 200 CFU/mL of *E. coli*. The detection signal is the measured height of the HRP reduction peak at -0.45 V. The signal is measured by drawing a baseline. V_G amplifies the signal. Figure S3 (b) shows the CVs of a negative control electrode. Only a weak current step appears at -0.45 V. Applying V_G does not affect the step.

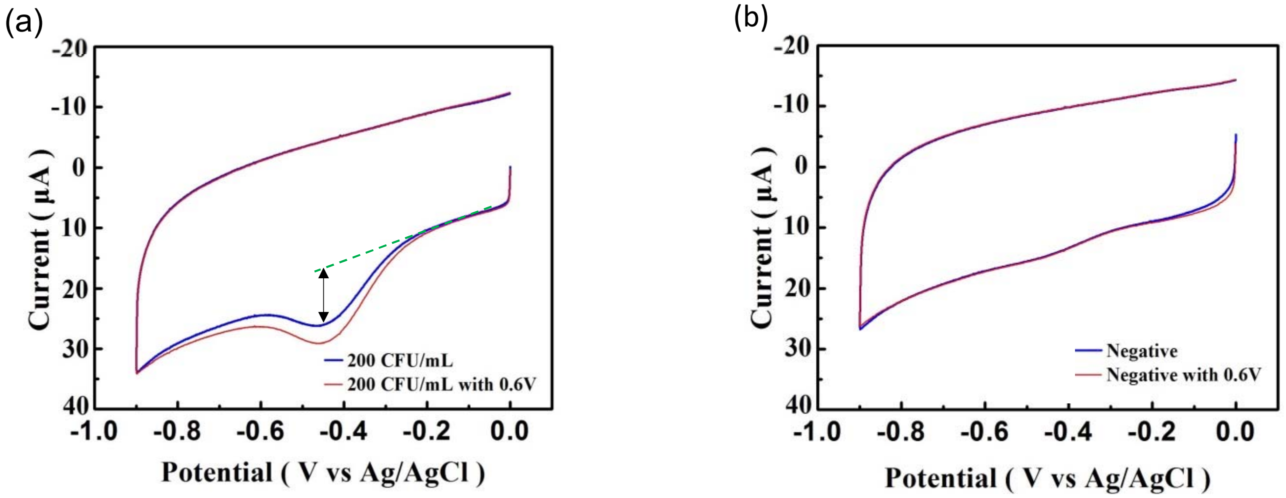

Figure S3 Characteristic CVs of detection electrodes with (a) 200 CFU/ml of *E. coli* with and without signal amplification or (b) no bacteria (negative control; NC) with and without amplification.

3. AST with initial bacterial load

Figures S4a and **S4b** show the AST results using 8 μg/mL of ampicillin obtained with WT and ampR *E. coli*, respectively with an initial bacterial concentrations of 500-600 CFU/mL. This indicates that the platform effectively performs AST even at high bacterial loads.

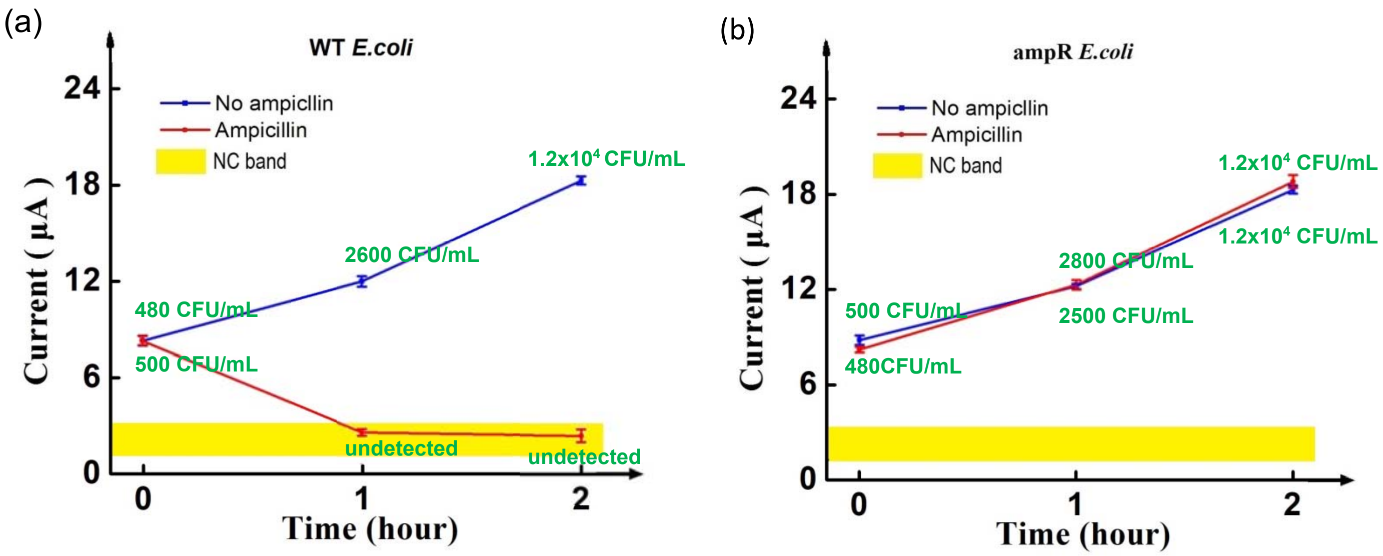

Figure S4 AST results using 8 μg/mL of ampicillin on (a) WT and (b) ampR *E. coli*, respectively. The initial bacterial concentrations were ~500 CFU/mL.

4. AST of *E. coli* MG1655

Figure S5 shows AST results on another susceptible strain of *E. coli* (MG1655; MIC 4μg/mL). The ampicillin-response of this strain is similar to that of WT *E. coli*. The results indicate the possibility of detecting different strains using one antibody system.

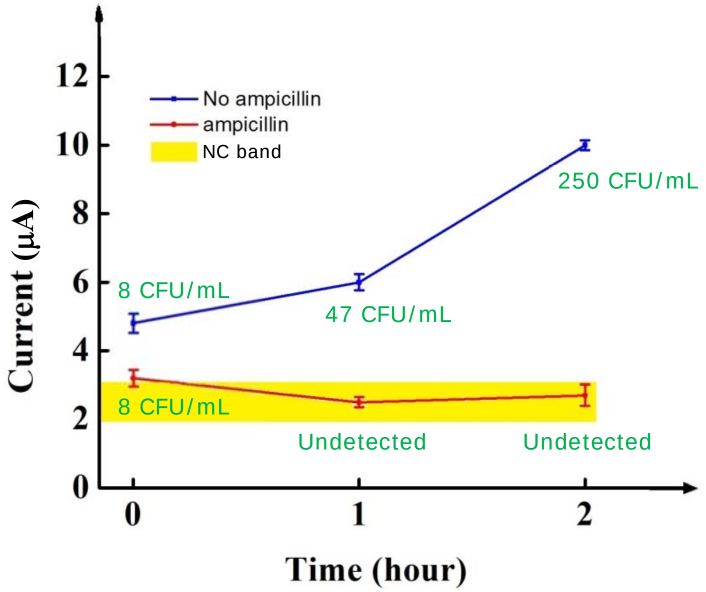

Figure S5 AST of the MG1655 strain of *E. coli*.

5. *E. coli* Transformation

A single colony of WT *E. coli* was added to 250ul transformation solution (50mM $CaCl_2$—BioRad 1660409) on ice and vortexed thoroughly. Then, 10ul pGLO™ plasmid (BioRad 1660405) was added to the suspension and kept on ice for 10 minutes. A negative control with no plasmid was maintained with similar conditions. Tubes were heat shocked in a 42°C water bath for 2 mins and rested on ice for 5 minutes. Then 250ul of tryptic soy broth + 1% glucose w/v (TSBG) was added and tubes were incubated overnight at room temperature. Samples were plated on 100ug/mL ampicillin (BioRad 1660407) and 500ug/mL arabinose (BioRad 1660406) tryptic soy agar (TSA) plates for colony selection. Single colony inoculates were grown under aerobic conditions in 100ug/mL ampicillin and 500ug/mL arabinose TSBG, and a 25% glycerol stock of the mid-log (OD_{600} = 0.50-0.55) cultures was maintained at −70 °C. Successful transformation of the plasmid and

resulting ampicillin resistance was confirmed by plating plasmid positive and negative control samples on ager with and without ampicillin (**Figure S6**). Since this plasmid also contains GFP under an arabinose promoter we confirmed transformation by confocal microscopy in the presence or absence of arabinose (**Figure S7**).

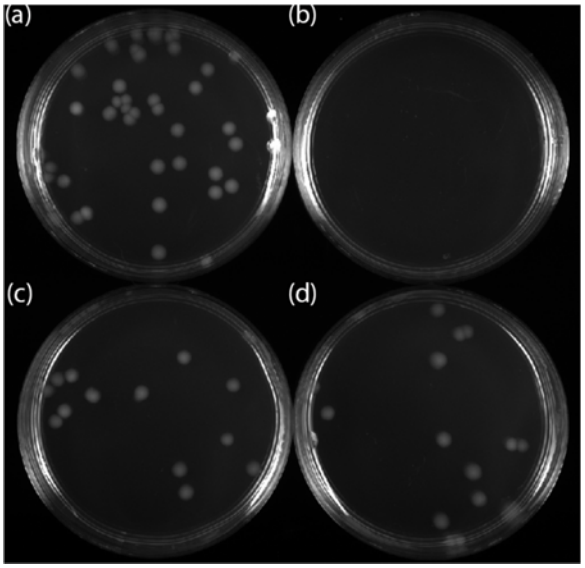

Figure S6 Confirmation of ampicillin resistance. (a) Negative plasmid control grown on un-supplemented and (b) TSA+100µg/ml ampicillin plates. (c) *E. coli* transformed with pGLO plasmid grown on un-supplemented and (d) TSA+100µg/ml ampicillin plates.

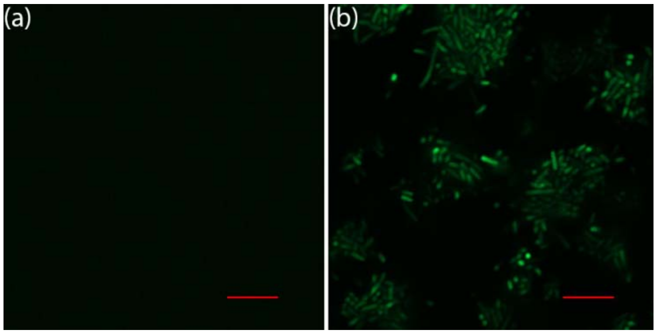

Figure S7 Confirmation of GFP expression. Confocal microscopy of *E. coli* transformed with pGLO plasmid grown (a) without and (b) with arabinose supplementation. Scale bar represents 10µm.

6. References

1. Choi, Y. & Yau, S.-T. A Field-Effect Enzymatic Amplifying Detector with Pico-Molar Detection Limit. *Analytical Chemistry* **81**, 7123-7126 (2009).

2. Csoregi, E., Gaspar, S., Niculescu, M., Mattiasson, B. & Schuhmann, W. (eds. Cuyper, M.D. & Bulte, J.W.M.) 105 (Kluwer Academic Publishers, Dordrecht, Boston, London, 2001).

3. Tans, S.J., Verschueren, A.R.M. & Dekker, C. Room-temperature transistor based on a single carbon nanotube. *Nature* **393**, 49-52 (1998).

4. Yau, S.-T. & Qian, G. A prototype protein field-effect transistor. *Applied Physics Letters* **86**, 103508 (2005).
